# The Evolving Scenario in the Assessment of Radiological Response for Hepatocellular Carcinoma in the Era of Immunotherapy: Strengths and Weaknesses of Surrogate Endpoints

**DOI:** 10.3390/biomedicines10112827

**Published:** 2022-11-06

**Authors:** Paolo Giuffrida, Ciro Celsa, Michela Antonucci, Marta Peri, Maria Vittoria Grassini, Gabriele Rancatore, Carmelo Marco Giacchetto, Roberto Cannella, Lorena Incorvaia, Lidia Rita Corsini, Piera Morana, Claudia La Mantia, Giuseppe Badalamenti, Giuseppe Brancatelli, Calogero Cammà, Giuseppe Cabibbo

**Affiliations:** 1 Section of Gastroenterology & Hepatology, Department of Health Promotion Sciences Maternal and Infant Care, Internal Medicine and Medical Specialties, PROMISE, University of Palermo, 90127 Palermo, Italy; 2 Department of Surgical, Oncological, and Oral Sciences (Di.Chir.On.S.), University of Palermo, 90127 Palermo, Italy; 3 Section of Radiology, Department of Biomedicine, Neuroscience and Advanced Diagnostics (BiND), University of Palermo, 90127 Palermo, Italy

**Keywords:** hepatocellular carcinoma, HCC, systemic therapy, immunotherapy, endpoints, radiological criteria, RECIST 1.1, mRECIST

## Abstract

Hepatocellular carcinoma (HCC) is a challenging malignancy characterised by clinical and biological heterogeneity, independent of the stage. Despite the application of surveillance programs, a substantial proportion of patients are diagnosed at advanced stages when curative treatments are no longer available. The landscape of systemic therapies has been rapidly growing over the last decade, and the advent of immune-checkpoint inhibitors (ICIs) has changed the paradigm of systemic treatments. The coexistence of the tumour with underlying cirrhosis exposes patients with HCC to competing events related to tumour progression and/or hepatic decompensation. Therefore, it is relevant to adopt proper clinical endpoints to assess the extent of treatment benefit. While overall survival (OS) is the most accepted endpoint for phase III randomised controlled trials (RCTs) and drug approval, it is affected by many limitations. To overcome these limits, several clinical and radiological outcomes have been used. For instance, progression-free survival (PFS) is a useful endpoint to evaluate the benefit of sequential treatments, since it is not influenced by post-progression treatments, unlike OS. Moreover, radiological endpoints such as time to progression (TTP) and objective response rate (ORR) are frequently adopted. Nevertheless, the surrogacy between these endpoints and OS in the setting of unresectable HCC (uHCC) remains uncertain. Since most of the surrogate endpoints are radiology-based (e.g., PFS, TTP, ORR), the use of standardised tools is crucial for the evaluation of radiological response. The optimal way to assess the radiological response has been widely debated, and many criteria have been proposed over the years. Furthermore, none of the criteria have been validated for immunotherapy in advanced HCC. The coexistence of the underlying chronic liver disease and the access to several lines of treatments highlight the urgent need to capture early clinical benefit and the need for standardised radiological criteria to assess cancer response when using ICIs in mono- or combination therapies. Here, we review the most commonly used clinical and radiological endpoints for trial design, as well as their surrogacy with OS. We also review the criteria for radiological response to treatments for HCC, analysing the major issues and the potential future perspectives.

## 1. Introduction

Hepatocellular carcinoma (HCC) is the most common primary liver cancer, and it represents the fifth most common cancer and the second most frequent cause of cancer-associated death worldwide [1]. Chronic infection with hepatitis B virus (HBV) is the predominant risk factor for HCC in Southeast Asia and Africa, while chronic infection with hepatitis C virus (HCV) is the predominant risk factor for HCC in Western countries and Japan. However, the incidence of HCC related to HBV or HCV infection is decreasing in developed countries, and it is counterbalanced by the increasing prevalence of non-alcoholic fatty liver disease (NAFLD), obesity, and type 2 diabetes [2,3,4]. HCC is a challenging malignancy of global importance and is characterised by wide clinical and biological heterogeneity in its early, intermediate, and advanced stages [5,6,7], as well as a dismal prognosis [8]. The complex management of HCC in both the early and advanced stages of the disease, along with its coexistence with chronic liver disease, requires a multidisciplinary approach [9,10] in order to offer the best treatment for each patient in terms of benefit, risks, and costs. Curative treatments for early-stage HCC include surgical resection, percutaneous ablation, and liver transplantation (LT). Unfortunately, despite the application of surveillance programs [11], many patients have disease that is not amenable to curative treatments at presentation, and for this reason they undergo treatments such as transarterial chemoembolisation (TACE), radioembolisation (TARE), and systemic treatment [12,13]. Particularly, the landscape of systemic treatments has rapidly evolved in recent years. Following the approval of sorafenib in 2008 [14], no further effective systemic therapy options were identified for almost a decade. In recent years, however, several newer systemic therapy options with different mechanisms of action have shown efficacy in the first- and second-line settings [15,16,17]. Finally, the advent of immune-checkpoint inhibitors (ICIs) has had a dramatic impact on clinical decision-making for patients with unresectable HCC, showing survival and response rates rarely seen with previous systemic treatments [18,19]. Moreover, these treatments can reduce the tumour burden in a subgroup of patients, allowing effective downstaging and increasing the feasibility and the effectiveness of potential curative options that were initially discarded [20]. The advent of new effective systemic therapies requires a careful clinical reflection on which endpoints to use in randomised controlled trials (RCTs) or in clinical practice to accurately measure the benefits and risks related to treatment.

The main goal of each oncological treatment is the improvement of overall survival (OS). Given that tumour progression is a major cause of death in cancer patients, tumour volume containment is crucial. However, HCC often arises in the setting of cirrhosis, which increases the risk of developing liver-related events that are not related to cancer, such as decompensation [21]. The coexistence of the tumour and cirrhosis plays a critical role in designing clinical trials and using proper clinical endpoints. While OS is the most accepted endpoint for phase III RCTs, it is conditioned by post-progression treatments and crossover bias. To overcome these limits, several clinical and radiological endpoints have been used to determine drug approval, although the surrogacy between these endpoints and OS remains to be validated in the setting of unresectable HCC.

**Figure 1:**
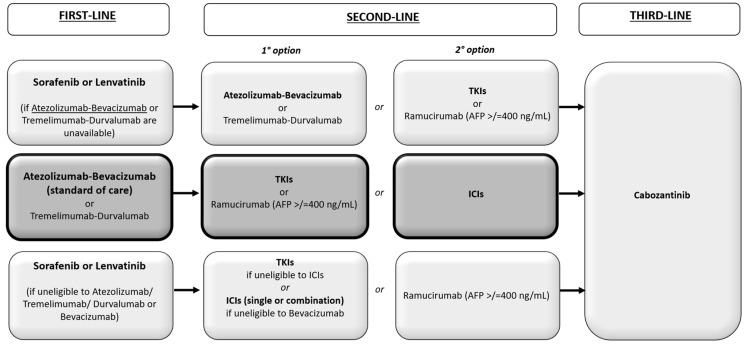
Therapeutic algorithm of systemic therapies proposed by the Italian Association of Liver Diseases (AISF) [29].

Since the reduction of the tumour burden or the complete response after therapy could affect OS and progression-free survival (PFS), the evaluation of radiological tumour response during treatment plays a critical role in the management of patients [22]. However, some treatments for HCC, such as locoregional therapies—particularly TACE and TARE—can induce tumour necrosis without appreciable changes in size. Therefore, relying only on dimensional imaging criteria may not allow a correct evaluation of response to treatment in patients with HCC. Furthermore, the recent introduction of new effective systemic therapies for advanced stages—including those based on immunotherapy—further increased the complexity in the evaluation of the response. In fact, the infiltration of immune cells into the tumour via the checkpoint inhibitor (CI) can cause an increase in the diameter of the tumour that could initially meet the criteria of disease progression (PD), without implying a true progression (so-called pseudo-progression) [23,24]. Moreover, most recent first-line clinical trials were compared to sorafenib, and all of the second-line therapies were designed when sorafenib was the standard of care. Hence, it is not clear which sequential treatment is most effective [25]. Several societies have proposed therapeutic algorithms for systemic therapies [26,27,28]. Figure 1 shows the algorithm proposed by the Italian Association of Liver Diseases (AISF) [29]. Hence, a standardised approach to assess response to treatment and the identification of surrogate endpoints useful for the early prediction of OS are urgently needed in this setting. In this review, we describe the most commonly used clinical and radiological endpoints for trial design, along with their surrogacy with OS. We also review the criteria for radiological response to treatments for HCC, analysing the major issues and the possible future perspectives.

## 2. Search Strategy

Studies for review in this article were retrieved from the PubMed database using the search terms “hepatocellular carcinoma”, “liver cancer”, and “primary liver carcinoma”, both individually and in combination with the terms “radiological criteria”, “RECIST”, “EASL criteria“, “mRECIST”, “Li-RADS”, and “endpoint”. The search included literature published in English prior to October 2022.

## 3. Clinical and Radiological Endpoints in HCC

Overall, three kinds of endpoints have been defined in the setting of anticancer therapy:

(1) Solid hard endpoints, such as OS.
(2) Surrogate endpoints, such as progression-free survival (PFS), time to progression (TTP), and objective response rate (ORR).
(3) Patient-reported outcomes, such as quality of life (QoL) [30,31].

Table 1 summarises the main features of the most frequently used oncological endpoints.

### 3.1. Hard Endpoints

OS is defined as the time from the start of treatment (or randomisation) to death, and it has been used as an endpoint for drug approval, being a universally recognised, easy-to-assess endpoint to determine clinical benefit in oncology trials [32]. Although it represents the most robust endpoint to identify a drug benefit, a long follow-up is required to assess the improvement in survival, and this leads to longer trials and slower drug approval. Moreover, OS does not take into account post-progression survival and treatment crossover [33], meaning that that OS is affected by the proportion of patients receiving second-line treatments after the first-line failure, as well as by their effectiveness. While this was not a relevant issue when only a few active drugs were available for advanced HCC, today it represents a matter of debate when evaluating the benefit of innovative treatments [30]. Moreover, the impact of post-progression therapies on the evaluation of OS could be a partial explanation for the improvement of outcomes of patients treated with sorafenib as a control arm in recent phase III RCTs [21,34].

### 3.2. Surrogate Endpoints

To overcome the limits of OS, surrogate endpoints have been used in cancer trials. A surrogate endpoint is defined as any measure used as a substitute for a clinically meaningful endpoint, with the ability to predict the net effect of the intervention [35], possibly in advance of hard endpoint. A surrogate endpoint needs to be validated at both the trial (i.e., by using aggregate data) and individual levels (i.e., by using individual patient data) [36]. Since surrogate endpoints require a smaller sample size than hard endpoints, and since they shorten the duration of studies, in recent years they have been used as primary endpoints in clinical trials to obtain fast-track drug approval [33,37]. However, the poorly demonstrated surrogacy between hard and surrogate endpoints is a matter of concern. In effect, lack of surrogacy could translate into the approval of ineffective or even harmful drugs. Surrogacy is not just a methodological dilemma in order to ensure proper trial design; it is also the key to guaranteeing the approval of effective drugs as rapidly as possible and, in the context of multiple treatments being available, to defining proper sequential treatment strategies [21,25].

The most commonly evaluated surrogate endpoints in the field of HCC are generally related to radiological tumour response, and they can be classified as time-dependent or time-independent endpoints, depending on whether or not they are able to capture the time to the event.

**Figure 2:**
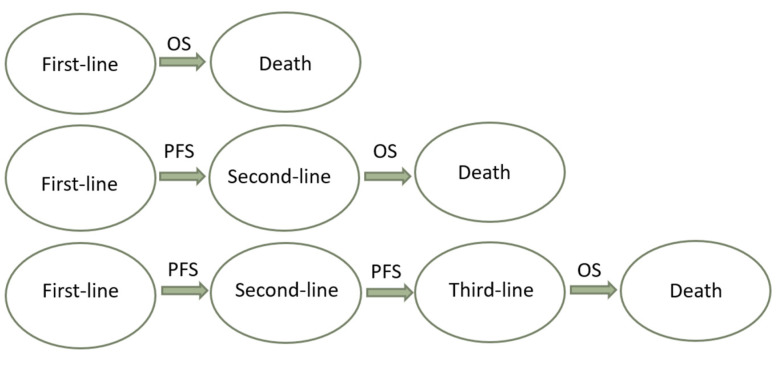
Evaluation of the benefit of sequential therapies: overall survival is the result of the sum of progression-free survival of different subsequent lines of therapy.

- Time-independent endpoints include objective response rate (ORR)—defined as the proportion of patients achieving complete (CR) and partial (PR) radiological responses—and disease control rate (DCR), which also includes stable disease. Radiology-based endpoints are able to capture early anticancer activity; hence, they are helpful in early-phase studies (i.e., phase I or II). A high ORR may also identify the treatments that are more suitable for downstaging purposes, meaning a stage migration toward the possibility of performing locoregional treatments. The main disadvantage of ORR and DCR is that they are not able to identify the time when the event occurs, and they are also subject to assessment bias. Indeed, the rate of radiological responses can be affected by both the subjectivity of the radiologists and the adopted radiological criteria.
- Time-dependent surrogate endpoints include time to recurrence (TTR) and recurrence-free survival (RFS) in the setting of curative treatments, and TTP and PFS in the setting of advanced HCC. TTR and TTP are defined as the time from the start of treatment (or randomisation) to radiological recurrence or progression, respectively. TTP has been adopted as a secondary endpoint in HCC trials [14]. Although TTP is not biased by post-progression treatments and it reports the time to the event, it is unable to detect important events such as death, and it is subject to the same assessment bias as ORR [38]. RFS and PFS are composite time-dependent endpoints, often used as both primary and secondary endpoints in oncological trials. RFS is defined as the time from the start of treatment (or randomisation) to radiological recurrence or death, and it is employed in curative settings (i.e., after surgery or ablation). PFS is defined as the time from the start of treatment to radiological progression or death, and it is commonly used in the setting of systemic treatments. These are not influenced by post-recurrence or post-progression treatments and crossover bias. For these reasons, PFS could be useful to evaluate the effectiveness of sequential therapies in settings where multiple treatment lines are available. Based on this assumption, the patient’s journey could be represented as the sum of multiple subsequent PFSs of sequential treatment lines (Figure 2) [21,25,39]. The use of PFS was initially discouraged in HCC trials due to the risk of competing events and the coexistence of cancer and underlying liver disease. This problem was mitigated by including only patients with well-preserved liver function in HCC trials [40]. Overall, PFS has been used to obtain accelerated drug approval in several oncological settings [37]. It has been estimated that between 2009 and 2014, 66% of anticancer drugs approved by the FDA were approved on the basis of PFS. Most anticancer trials also use PFS as a primary endpoint, and around 50% of them have shown benefits to OS [41]. The counterpart of this phenomenon is the possibility to introduce ineffective or harmful drugs to the market, which need to be evaluated with hard endpoints in post-marketing studies [42]. For instance, a critical review of anticancer drugs approved based on PFS is crucial—not only with regard to their efficacy, but also their safety. In fact, it has been speculated that poor drug tolerance may result in imbalanced dropouts of the experimental drug, and due to informative bias this might lead to longer PFS without any improvement of OS or quality of life [38]. Time to treatment failure (TTF), defined as the time from the start of treatment to its discontinuation for any reasons (e.g., death, progression, or toxicity), has been proposed to overcome this limitation of PFS, although it is not recommended by the FDA for drug approval. This issue should be widely considered when there is no surrogacy between PFS and OS in the context of HCC, and could be particularly relevant if a competing survival event—such as hepatic decompensation with respect to tumour progression—is not usually recorded by trials [29,43]. To this end, novel endpoints aiming to measure the time to hepatic decompensation or the decompensation-free survival are needed to improve the evaluation of the net benefit of systemic treatments for HCC. To summarise, the main limitations of surrogate endpoints are the assessment bias and the need for surrogacy validation. Assessment bias might be mitigated at the trial level by using central expert radiology reviews. Validation of a surrogate endpoint is related to the ability of that endpoint to quickly and accurately predict the clinical outcome. Validation needs to be performed at both the individual and trial levels, using individual patients’ data and aggregated data, respectively [33].

### 3.3. Validation of Surrogacy

Validated surrogate endpoints are needed in order to guarantee an accurate evaluation of treatment benefit in clinical practice and in RCTs [30]. Several studies and recent meta-analyses at both the individual and trial levels have tried to assess the correlation between PFS and OS in oncology and, more specifically, in the advanced HCC setting [44,45,46]. Llovet et al. published a recent systematic review identifying 21 studies of advanced HCC, considering both first- and second-line treatments. Hazard ratios (HRs) of TTP, PFS, and OS were used, and the overall correlation between OS and PFS was determined. A moderate Pearson’s correlation (R = 0.84) was found. Since trials with HRs of OS and PFS above 0.6 did not evidence any survival benefit, the authors also proposed a HR value < 0.6 as a surrogate threshold to define validation of PFS. Afterwards, six additional phase III RCTs including anti-PD-1 agents were analysed. Two of them were positive studies for OS and showed an HR < 0.6 for PFS. The remaining four trials were negative for survival, and all of them had an HR > 0.6 for PFS [47]. Some limitations of these studies are that they did not include immunotherapy agents and, more importantly, the use of HRs as a comparative measure requires the proportionality of hazard over the entire follow-up period [48]. To overcome this problem, we used innovative methods to investigate whether PFS is a surrogate of OS, by using individual patient survival data extracted from Kaplan–Meier curves. In a meta-analysis including 49 studies—11 of them assessing ICIs and 38 trials assessing tyrosine kinase inhibitors (TKIs)—non-proportionality of HR was present in two-thirds of TKI trials. As the HRs were not proportional, median times, time-based endpoints (first and third quartiles), and restricted mean survival time (RMST) were used to assess the surrogacy of PFS and OS. Surrogacy between PFS and OS evaluated by R^2^ was 0.89 and 0.50 in ICIs and TKIs, respectively. According to this study, a high surrogacy was identified with ICIs but not with TKIs for HCC [46]. The advent of immunotherapy as a first-line treatment for uHCC [18,49] has increased the complexity of choosing the most appropriate endpoint in clinical trials. Consequently, in a context where more lines of treatment are available, OS represents the sum between PFS and post-progression survival (PPS). Generally, the longer the PPS, the lower the likelihood of PFS being correlated with OS. Since a long-term effect of ICIs has been demonstrated, PFS might be unable to capture the health benefits of immunotherapy [50,51]. Moreover, the mechanism of action of ICIs, which may involve delayed responses or long-term survival benefits in a subgroup of patients, could violate the assumption of the proportionality of HR, making it necessary to use new measures to evaluate treatment benefits, including RMST, milestone analysis, or accelerated failure time models [52]. Time to progression is also a well-recognised endpoint in oncology. It is generally used in early-phase trials to assess drug activity. Whether TTP correlates with OS is unclear, and its surrogacy has recently been studied in patients treated with TACE [53] and with TKIs [54,55]. A meta-analysis of nine TKI phase III trials found an unsatisfying surrogacy between TTP and OS, with R^2^ = 0.57. A higher surrogacy was found in the second-line studies, with R^2^ = 0.80 [54]. Afterward, Terashima et al. performed a systematic review and meta-analysis of 24 phase II and phase III studies, showing a low correlation between median OS and median TTP (R = 0.50) [55]. According to these findings, the use of TTP as the sole endpoint might be misleading. Similarly, moderate correlation of OS and TTP was also found in studies including patients treated with TACE [53,56].

In order to assess whether ORR is a valid surrogate of OS, Lencioni et al. performed an individual patient data analysis of the BRISK-PS trial [57]. BRISK-PS [58] was a negative randomised controlled phase III trial that included 395 patients with advanced HCC randomly assigned to receive the TKI brivanib or a placebo. The ORR was 11.5% (*n* = 26/226) with brivanib and 1.9% (*n* = 2/108) with the placebo. The OS of patients achieving an objective response was 15 months (95% CI 13.7–16.3), compared to those who did not have any objective response, with an OS of 9.4 months (95% CI 8.2–10.6) (HR = 0.28; 95% CI 0.14–0.54, *p* < 0.001). ORR was found to be an independent prognostic factor of OS in multivariate analysis. Moreover, the correlation of HRs for OS and ORR was high (R = 0.80; 95% CI 1–0.23, *p* = 0.091). Interestingly, the median time to objective response was 1.4 months, suggesting that ORR might be used as an early surrogate endpoint. More recently, a systematic review and meta-analysis studied the correlation between the HR of OS and the odds ratio of objective response, comparing mRECIST and RECIST at the trial level. A modest surrogacy was found between OS and ORR in 11 RCTs where the mRECIST criteria were used; the R was 0.677 (95% CI 0.655–0.697; *p* = 0.022), suggesting a positive correlation. Kudo et al. also assessed 29 studies where the RECIST criteria were used. The correlation of ORR and OS was even lower with this set of criteria, showing an R of 0.532 (95% CI 0.519–0.545; *p* = 0.003). A direct comparison between the six trials where both mRECIST and RECIST were assessed showed a stronger correlation of the former criteria (R = 0.707; 95% CI 0.685–0.728; *p* = 0.116 vs. R = 0.622; 95% CI 0.593–0.649; *p* = 0.187) [59]. The same authors performed a meta-analysis of five studies in order to evaluate the impact of mRECIST-related ORR on OS. A pooled HR of 0.44 (95% CI 0.27–0.70; *p* < 0.001) was found, indicating improved clinical outcomes in responders versus non-responders. Hence, ORR could be considered an independent predictor of OS [59]. Although more evidence must still be gathered before recommendations can be made, [60] ORR could be used as a primary endpoint for phase II trials.

### 3.4. Radiology

**Figure 3:**
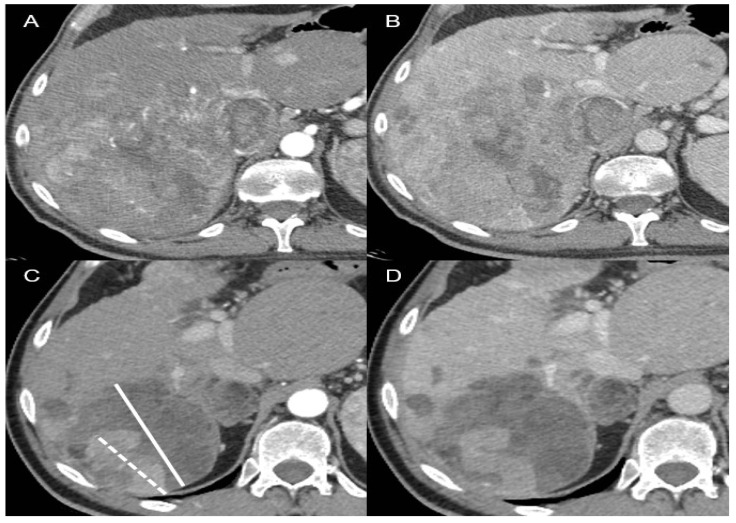
A 61-year-old man with infiltrative hepatocellular carcinoma and tumour thrombosis of the inferior vena cava: On baseline contrast-enhanced CT ((**A**), arterial phase; (**B**), portal venous phase), the largest tumour diameter measures 12.8 cm. CT follow-up ((**C**), arterial phase; (**D**), portal venous phase) after three months of atezolizumab–bevacizumab demonstrates a decrease in the size of the lesion, with a total tumour diameter of 10.8 cm (continuous line), consistent with stable disease according to the RECIST 1.1 criteria, but with a residual internal enhancing component of 6.1 cm (dashed line), consistent with a partial response according to the mRECIST criteria.

Since the most commonly used surrogate endpoints are based on radiological assessment (i.e., PFS, TTP, ORR), it is important to understand which are the features of the most commonly employed radiological criteria, both in clinical practice and in RCTs [61,62]. Ideally, cancer-related death is associated with progression in the size of the tumour and the spread of metastases. On the other hand, the goal of any cytotoxic drug is to reduce the tumour mass. Therefore, the different radiological criteria aim to assess the changes in tumour burden. This might not be sufficient in the HCC setting, particularly in the setting of systemic therapies. As discussed later in this paper, treatment with TKIs is associated with a lower probability of obtaining a neoplastic shrinkage, and the other ICIs may have unconventional patterns of radiological response, including delayed responses or pseudo-progression, manifesting first in enlargement of the tumour burden and later in radiological shrinkage [63]. Moreover, the different types of progressive disease have been found to modify the overall survival [64]. It has been shown that patients with new extrahepatic lesions or vascular invasion have a worse prognosis compared to patients whose progression is defined by new intrahepatic lesions or by enlargement of pre-existing liver nodules, either under TKIs or immunotherapy [64,65]. In addition to the adopted response criteria, overall responses are categorised as complete response (CR), partial response (PR), stable disease (SD), and progressive disease (PD), given a predefined threshold of dimensional change. The criteria differ from one another in terms of the threshold to define PR or PD, detection of the viability of the tumour, and whether they are unidimensional or bidimensional measurements. The RECIST 1.1 criteria are the globally accepted criteria in oncology. They are based on evidence derived from traditional chemotherapy studies, and they assess the response of up to five target lesions and up to two organ lesions. The maximum diameter of every target lesion is measured. The viability of the tumours is not taken into account, and they are unidimensional [66]. Some weaknesses of RECIST 1.1 include the inability to detect tumour necrosis or viability; therefore, treated lesions without arterialisation would count as SD instead of CR or PR. Due to these limitations, the EASL criteria have been proposed. Novelties of this system include bidimensionality and the fact that they can differentiate viable and non-viable tissue. Only lesions with classic arterial enhancement and subsequent washout might be considered to be active HCC [67]. In 2010, Llovet and Lencioni provided a modified version of the response evaluation criteria in solid tumours (mRECIST). This new set of criteria are unidimensional, and target lesions are considered when they have arterial enhancement, although the dimensional thresholds are similar to those of RECIST. New specifications for confounders such as ascites and lymph nodes were developed [61]. Figure 3 outlines different treatment responses according to RECIST 1.1 and mRECIST after immunotherapy. Moreover, the combination of more than one radiological criterion has been investigated to assess response to locoregional treatments (LRTs). Riaz et al. combined morphological criteria assessing tumour size (i.e., RECIST and WHO) with EASL criteria, where they evaluated tumour necrosis according to enhancement reduction. They compared the receiver operating characteristics (ROCs) of each criterion and their combination after LRTs. While combining the EASL and WHO criteria was slightly more accurate (AUC 0.85), the assessment of enhancement to predict tumour necrosis with the EASL criteria (AUC 0.82) far exceeded the accuracy of morphological evaluation (WHO AUC 0.68) [68].

**Figure 4:**
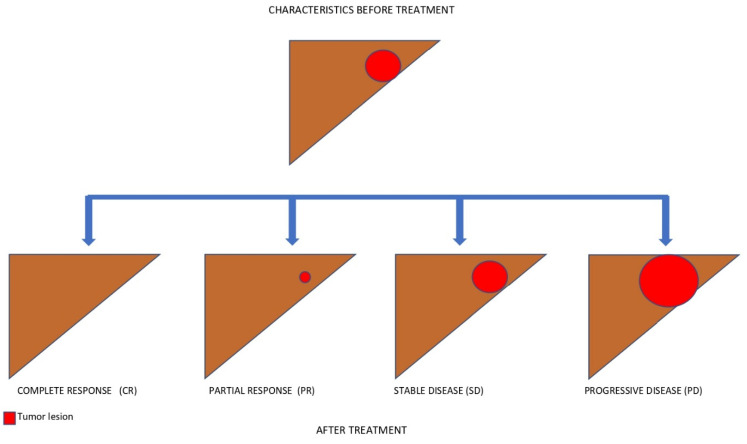
Radiological response according to RECIST 1.1.

**Figure 5:**
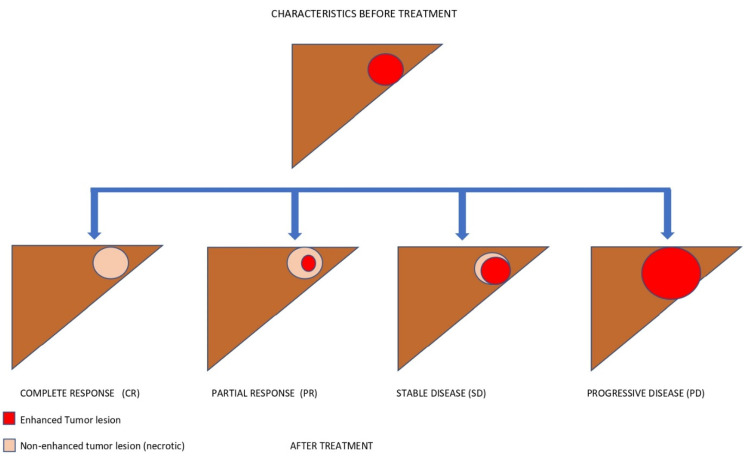
Radiological response according to mRECIST.

Finally, the Japan Society of Hepatology has proposed the Response Evaluation Criteria in Cancer of the Liver (RECICL), now in its sixth version. Although the RECICL criteria take into account the viability of tumour with both the arterial and portal phase, use a bidirectional measurement approach, and have also been studied to assess responses to locoregional treatments and to systemic therapies, they are not widely used in clinical trials [69]. Table 2 summarises the main features of the radiological criteria to assess responses to systemic treatments for HCC. Figure 4 and Figure 5 show radiological responses according to RECIST 1.1 and mRECIST, respectively. As previously stated, ICI-based combination treatments have now become the new standard of therapy for uHCC [18,29,49]. The impact of ICIs on radiological assessment has been studied in other cancer histotypes, and specific criteria of response to immunotherapy have been proposed [70], known as immunoRECIST. This set of criteria takes into account the risk of pseudo-progression, including the unconfirmed progressive disease (iUPD) and confirmed progressive disease (iCPD). Indeed, since the response to immunotherapy might be delayed, before defining an increase in tumour size as tumour progression, this needs to be confirmed after 4–8 weeks from the iUPD. However, the immunoRECIST criteria have not yet been validated for HCC, and they need more studies in order to be standardised. In the near future, it is likely that artificial intelligence (AI) and radiomics will help clinicians and radiologists to assess radiological response more accurately and to predict treatment response at earlier phases after either locoregional treatments or systemic therapies [71]. Radiological criteria need to reflect the biological effect of a treatment and, moreover, to be correlated with survival. Since mRECIST and RECICL have the ability to detect tumour viability, they are considered to be superior for assessing response after locoregional treatments [72,73,74]. This becomes less clear in the setting of systemic therapies. The inadequacy of RECIST 1.1 was suggested after the first trials of sorafenib [14]. In these trials, sorafenib was shown to increase OS and PFS, although the ORR was very low. In the SHARP trial, according to the RECIST criteria, 2 of 299 patients had PR and 71 of 299 patients had SD in the sorafenib group. The DCR was higher in patients treated with sorafenib compared to those treated with a placebo [14]. Similar results were found in the Asia-Pacific trial, in which only 5/150 (3.3%) patients had a partial response, while 81/150 (54%) had stable disease in the sorafenib group, and no complete response was registered according to the RECIST 1.1 [75]. Since then, a series of studies have tried to assess whether the mRECIST criteria are superior to RECIST 1.1 in patients treated with TKIs. First, Edeline et al. found that among 53 patients treated with sorafenib, the ORR was 2% and 23% according to RECIST and mRECIST, respectively. However, of the 42 patients classified as SD with RECIST 1.1, 11 were classified as PR with mRECIST. The survival of patients achieving an objective response according to mRECIST was superior to that of those who did not respond [76]. Similar results were found by Ogasawara et al. [77]. The inability of RECIST 1.1 to detect PR has also been confirmed by Takada et al., in a study of 191 patients with uHCC treated with sorafenib. Similar to previous studies, the ORR was higher according to mRECIST than RECIST 1.1, although the DCR did not change substantially [78]. In 2018, the REFLECT trial demonstrated the non-inferiority of lenvatinib compared to sorafenib [15]. Radiological endpoints were assessed with both RECIST 1.1 and mRECIST. The ORR was significantly better with lenvatinib compared to sorafenib. Moreover, substantial differences were found among the adopted criteria; indeed, mRECIST showed a greater ORR (40·6%, 36·2–45·0) compared to RECIST 1.1 (18·8%, 15·3–22·3). As expected, 184 (38%) patients were defined as PR with mRECIST vs. 88 (18%) with RECIST 1.1. A subsequent analysis of the REFLECT trial found that ORR was also an independent predictor of OS, regardless of the treatment (HR 0.61 [0.49–0.76] *p* < 0.001) [79]. The higher response rate of lenvatinib [80] compared to sorafenib in a real-life study also has major clinical implications [81]. It has been speculated that achieving a higher tumour response could lead to longer PFS, and this might help clinicians in using a sequential treatment of systemic therapies [21,25]. The relatively high response and DCR of lenvatinib has been confirmed in a real-world study that also analysed post-lenvatinib treatments. This study outlined a median OS of 47 months when sequencing immunotherapy after lenvatinib. This long survival raises questions as to the best time to switch from locoregional treatment to systemic therapies, as well as the impact of first-line treatments able to achieve tumour response [82]. In a recent post hoc analysis of the REFLECT trial, authors investigated the relationship of ORR as determined by mRECIST and RECIST 1.1 with OS. Patients were defined as responders if they achieved PR or CR. Non-responders were those patients who had SD or PD. OS was estimated for responders and non-responders. Moreover, landmark analyses of OS by ORR status were conducted at 2, 4, and 6 months. Responders achieved 21.6 (95% CI: 18.6–14 24.5) months of survival; non-responders had a median OS of 11.9 months (95% CI: 10.7–12.8). OS was higher in responders compared to non-responders irrespective of whether the mRECIST or RECIST 1.1 criteria were used. Interestingly, the OS benefit of patients who achieved a radiological response according to mRECIST was maintained at each landmark timepoint. ORR according to mRECIST was also found to be an independent predictor of OS by multivariate analysis, independently of the treatment arm (lenvatinib or sorafenib). Thus, the authors suggested that ORR is associated with OS at the individual level [83,84]. Although new evidence is emerging for the potential surrogacy of ORR and OS in systemic therapies for HCC, these studies are confined to tyrosine kinase inhibitors, and it is not known whether the same surrogacy level exists for other therapies, such as immunotherapy, or whether RECIST 1.1 or mRECIST represent the best evaluation response criteria for ICIs. Indeed, the combination of atezolizumab plus bevacizumab proved to be more effective than sorafenib in the phase III IMBrave150 trial [18], changing the paradigm of the treatment of unresectable HCC and establishing atezolizumab–bevacizumab as the new standard of care. The combination of atezolizumab plus bevacizumab reached an impressive OS of 19.2 months compared to the OS of the sorafenib group (13.4 months). The median PFS of atezolizumab–bevacizumab was higher compared to sorafenib (6.8 months vs. 4.3 months. HR = 0.59; 95% CI 0.47–0.76; *p* < 0.001). According to the PFS, the ORR and DCR were subsequently tested with both RECIST 1.1 and HCC-specific mRECIST. The confirmed ORR was 27.3% in the atezolizumab–bevacizumab group and 12% in the sorafenib group according to RECIST 1.1 (*p* < 0.001). Eighteen patients (5.5%) achieved CR and 71 patients (21.8%) had PR with atezolizumab and bevacizumab; no patients in the sorafenib group had a complete response. Compared to RECIST 1.1, mRECIST showed a higher ORR of 33.2% with immunotherapy vs. 13.3 with sorafenib (*p* < 0.001). Using mRECIST, 33 patients with atezolizumab–bevacizumab had a complete response (10.2%). The duration of treatment responses longer than 6 months was similar, independent of the radiological criteria. In the recent HIMALAYA trial—a phase III study—a regimen of anti-CTLA-4 (cytotoxic T-lymphocyte antigen 4) tremelimumab plus the anti-PD-L1 durvalumab was compared with sorafenib and with durvalumab monotherapy. The STRIDE regimen (a single priming dose of tremelimumab added to durvalumab) was shown to increase OS compared to sorafenib. The median OS of the STRIDE regimen was 16.4 months, compared to 13.7 months with sorafenib. No differences in PFS were observed between the two groups. Radiological response was assessed by using the RECIST 1.1 criteria. Twelve patients (3.1%) in the STRIDE group achieved a CR; no radiological response was observed with sorafenib. The DCR was similar between the groups [49]. The most recent trial of immunotherapy for advanced HCC evaluated the combination of cabozantinib plus atezolizumab versus sorafenib as first-line treatments [19]. The study included 837 patients with unresectable HCC that had not been previously treated. No significant differences in OS were found at the interim analysis. A longer PFS of atezolizumab–cabozantinib compared with sorafenib was found, with 6.8 months (99% CI 5.6–8.3) in the combination treatment group versus 4.2 months (2.8–7.0) in the sorafenib group (HR 0.63, 99% CI 0.44–0.91, *p* = 0.0012). Additionally, in this trial, radiological responses were assessed according to the RECIST 1.1 criteria. In the intention-to-treat population, the combination of atezolizumab–cabozantinib showed an ORR of 11%, compared to 4% for sorafenib. Only one CR was observed in the combination group. The DCR was comparable between the two arms—78% with atezolizumab–cabozantinib and 65% with sorafenib. Table 3 shows the findings of radiological endpoints (i.e., ORR and PFS) according to RECIST 1.1 and mRECIST in most relevant RCTs evaluating systemic therapies for uHCC.

## 4. The Relevance of Liver Function

In the majority of cases, HCC arises in patients with advanced chronic liver disease. Hence, liver function plays a crucial role in the feasibility and safety of HCC treatments at all stages. It has largely been shown that the treatment of the aetiological factors of liver disease prevents hepatic decompensation, reduces liver-related mortality, and prolongs survival [90]. HCV is still the main cause of liver cirrhosis in Western countries. Since the use of direct-acting antivirals (DAAs) has significantly increased the rate of sustained virologic response (SVR) and the curing of HCV, their effect on the occurrence and recurrence of HCC has been studied extensively [90,91]. DAAs not only reduce the risk of hepatic decompensation, widening the access to curative treatments in patients with early HCC, but their benefit might also be maintained in patients undergoing systemic therapies. It has also been largely demonstrated at the individual patient level that the use of DAAs is not associated with higher recurrence of HCC [92], disavowing some initial warning signals. Similar evidence exists for HBV infection, with a well-known effect of nucleoside/nucleotide analogues in reducing the risk of hepatic decompensation and improving OS [93,94]. Unfortunately, to date, no pharmacological treatments have yet been approved for non-alcoholic steatohepatitis-related cirrhosis, although several drugs have shown promising results in phase II and III RCTs [95]. Since the preservation of liver function is a necessary condition for the effective and safe application of any active treatment for HCC, whether in the early or advanced stages, it is relevant when evaluating an innovative treatment (whether systemic, locoregional, or a combination of the two)—not only to assess the efficacy against cancer, but also to take into account the impact of the treatment on liver function. In the setting of advanced HCC, HCC progression and liver decompensation represent two competing events that can potentially lead to death independently from one another [43]. However, evolutionary events related to liver function—such as changes in Child–Pugh, MELD, or albumin–bilirubin (ALBI) scores, or the occurrence of liver-decompensating events, such as ascites, portal hypertensive bleeding, hepatic encephalopathy, or jaundice—are rarely reported in RCTs, and only real-world studies are able to report these data [96]. Therefore, endpoints related to deterioration of liver function should be routinely reported in RCTs, including the time to decompensation, which can be defined as the time from the start of treatment to hepatic decompensation; or decompensation-free survival—a composite endpoint defined as the time from the start of treatment to hepatic decompensation or death [29]. It is expected that the introduction of these novel endpoints could improve the interpretation of the effectiveness and safety of treatments for HCC, and it could also potentially explain the unsatisfactory surrogacy between radiology-based outcomes and OS.

## 5. Conclusions

The rapid growth of effective systemic treatments for HCC has highlighted the need for new surrogate endpoints to capture clinical benefit early, as well as the need for standardised radiological criteria to assess cancer response when using ICIs as mono- or combination therapies. PFS, TTP, and ORR are commonly used in oncology as radiology-based surrogate endpoints of OS to accelerate drug approval and to capture OS benefit early. However, in the HCC setting, the surrogacy between radiology-based endpoints and OS remains to be validated at the individual level—especially for recently approved combination treatments including ICIs. Moreover, the subjectivity of radiological assessment and the heterogeneity between different radiological criteria to assess response represent inherent limits when these endpoints are used to interpret the benefit of a treatment without considering hard endpoints (i.e., OS) or evolutionary clinical events related to the deterioration of liver function during follow-up. Nevertheless, PFS is a useful endpoint to evaluate the benefit of sequential treatments, since it is not influenced by post-progression treatments, unlike OS. In the rapidly evolving landscape of ICI-based combination treatments, the performance of ad hoc radiological criteria such as iRECIST should be further evaluated in the setting of HCC. Moreover, the unconventional patterns of response to ICIs raise the issue of testing the proportionality of HRs, which is not merely a methodological matter, but a clinically relevant issue for physicians and patients. Finally, we believe that a careful reporting of liver decompensation as a time-dependent event—both in RCTs and in real-world studies—is needed to improve the interpretability of the effectiveness and safety of novel treatments for HCC.

## Figures and Tables

**Table 1:** Endpoints in oncology trials.

Endpoint		Definition	Characteristics	Pros	Cons	
Hard Endpoints	OS	Time between random trial allocation and death from any cause	All regular FDA drug approvals for advanced HCC were based upon improvements in OS	Most robust endpoint in advanced HCC	Increasing number of effective therapies after progression → need for surrogate endpoints	
Surrogate Endpoints	ORR	The percentage of patients who achieve an objective tumour response		ORR, assessed by sensitive criteria, in single-arm phase II trials could be a useful tool to prioritise treatments for testing in phase III trials	The definition of duration of stable disease varies between studies	- Surrogate endpoints are vulnerable to interpretation bias (i.e., they rely on the radiological definition of tumour progression or response); - In order to be reliable, they require validation as credible predictors of OS
	TTP	The time between trial allocation and radiological progression, usually defined by standard criteria such as RECIST or mRECIST	Symmetric repeated radiological measurements every 6–8 weeks are required to avoid missing moderate differences between treatment groups	A moderate correlation has been established between PFS and OS	Not all types of tumour progression necessarily have the same clinical meaning (e.g., new extrahepatic/intrahepatic lesions, vascular invasion, growth of pre-existing lesions)	
	PFS	PFS is a composite endpoint of two variables—death, and evidence of radiological progression—usually defined by standard criteria such as RECIST or mRECIST		A moderate correlation has been established between PFS and OS	Competing risk of dying due to progressed liver dysfunction despite a relevant antitumor benefit	

**Table 2:** Radiological criteria to assess responses to systemic treatments for HCC according to RECIST 1.1, mRECIST, and iRECIST.

Parameters	Measurements of Lesions	Evaluated Parameters	Target Lesions (Max Number—Total)	Target Lesions (Max Number—per Organ)	Definition of CR	Definition of PR	Definition of PD	Definition of SD	Lymph Nodes	Criteria for Defining New Lesions
RECIST 1.1	Unidimensional	Total dimensions	5	2	Disappearance of all target lesions	≥30% decrease in the sum of diameters of target lesions	≥20% increase in the sum of diameters of target lesions	Any cases that do not qualify for either partial response or progressive disease	Considered as target lesions if short axis ≥15 mm	Unequivocal appearance
mRECIST	Unidimensional	Enhanced tumour	5	2	Disappearance of any intratumoral arterial enhancement in all target lesions	≥30% decrease in the sum of diameters of enhancing target lesions	≥20% in the sum of the diameters of enhancing target lesions	Any cases that do not qualify for either partial response or progressive disease	Porta hepatis lymph nodes: short axis ≥20 mm, all other locations ≥15 mm	Unequivocal appearance and typical HCC pattern
irRECIST	Unidimensional	Total dimensions	No change from RECIST 1.1	No change from RECIST 1.1	Resolution of all lesions, confirmed >4 weeks	Decrease of >30% in tumour burden in the absence of any new lesion or progression of non-target lesions	iUPD: Increase of > 20% in tumour burden from nadir; progression of NT lesions, or new lesions. iCPD: the imaging assessment performed 4–8 weeks after iUPD, confirms additional new lesions, further increase in previous new lesion size, or further increase in existing target or non-target lesions from iUPD	Clinical stability is considered when deciding whether treatment is continued after iUPD	No change from RECIST 1.1	to the same as in RECIST 1.1, but recorded separately and not included in the sum of lesions for target lesions identified at baseline

**Table 3:** Radiological endpoint assessment in RCTs of systemic treatment for unresectable HCC.

Trial	Treatment Arms	Patients (*n*)	ORR mRECIST (%)	ORR RECIST (%)	PFS mRECIST (Months)	PFS RECIST (Months)	TTP mRECIST (Months)	TTP RECIST (Months)
First Line								
SHARP [14]	Sorafenib	299	-	2.3	-	-	-	5.5
	Placebo	303	-	0.7	-	-	-	2.8
REFLECT [15]	Sorafenib	476	9.2	6.5	3.7	3.6	3.7	3.7
	Lenvatinib	478	24.1	18.8	7.4	7.3	8.9	7.4
IMBRAVE 150 [18]	Atezolizumab–bevacizumab	326	33.2	27.3	-	6.8	-	-
	Sorafenib	159	13.3	12	-	4.3	-	-
HIMALAYA [49]	Durvalumab–tremelimumab (STRIDE)	393	-	20	-	3.8	-	5.4
	Durvalumab	389	-	17	-	3.6	-	3.8
	Sorafenib	389	-	5	-	4	-	5.6
COSMIC 312 [19]	Cabozantinib–atezolizumab	432	-	11	-	6.8	-	7
	Sorafenib	217	-	4	-	4.2	-	4.6
	Cabozantinib	188	-	6	-	5.8	-	6.8
Checkmate 459 [85]	Nivolumab	371	-	15	-	3.7	-	3.8
	Sorafenib	372	-	7	-	3.8	-	3.9
Second Line								
RESORCE [16]	Regorafenib	379	11	7	3.1	3.4	3.2	3.9
	Placebo	194	4	3	1.5	1.5	1.5	1.5
CELESTIAL [17]	Cabozantinib	470	-	4	-	5.2	-	5.4
	Placebo	237	-	0.4	-	1.9	-	1.9
REACH-2 [86]	Ramucirumab	197	-	5	-	2.8	-	3
	Placebo	95	-	1	-	1.6	-	1.6
Checkmate -040 [87]	Nivolumab	214	-	20	-	4	-	-
Keynote 240 [88]	Pembrolizumab	278	-	18.3	-	3	-	3.8
	Placebo	135	-	4.4	-	2.8	-	2.8
Checkmate 040 [89]	Nivolumab–ipilimumab (arm A)	50	-	32	-		-	

## Data Availability

Not applicable.
